# In–Depth Characterization of Viral Isolates from Plasma and Cells Compared with Plasma Circulating Quasispecies in Early HIV-1 Infection

**DOI:** 10.1371/journal.pone.0032714

**Published:** 2012-02-29

**Authors:** Judith Dalmau, Francisco M. Codoñer, Itziar Erkizia, Maria Pino, Christian Pou, Roger Paredes, Bonaventura Clotet, Javier Martinez-Picado, Julia G. Prado

**Affiliations:** 1 AIDS Research Institute IrsiCaixa, Hospital Universitari Germans Trias i Pujol, Badalona, Barcelona, Spain; 2 Lifesequencing SL, Parc Cientific Universitat de Valencia, Paterna, Valencia, Spain; 3 Institució Catalana de Recerca i Estudis Avançats (ICREA), Barcelona, Spain; 4 Fundació Lluita contra la SIDA, Hospital Universitari Germans Trias i Pujol, Badalona, Barcelona, Spain; INSERM, France

## Abstract

**Background:**

The use of *in vitro* models to unravel the phenotypic characteristics of circulating viral variants is key to understanding HIV-1 pathogenesis but limited by the availability of primary viral isolates from biological samples. However, overall *in vivo* genetic variability of HIV-1 within a subject may not be reflected in the viable viral population obtained after isolation. Although several studies have tried to determine whether viral populations expanded *in vitro* are representative of *in vivo* findings, the answer remains unclear due to the reduced number of clonal sequences analyzed or samples compared. In order to overcome previous experimental limitations, here we applied Deep Pyrosequencing (DPS) technology in combination with phenotypic experiments to analyze and compare with unprecedented detail the composition of viral isolates and *in vivo* quasispecies.

**Methodology/Principal Findings:**

We amplified by DPS HIV-1 genomic regions covering *gag*, protease, integrase and *env*-V3 to characterize paired isolates from plasma and peripheral blood mononuclear cells and compare them with total plasma viral RNA in four recently HIV-1 infected subjects. Our study demonstrated the presence of unique haplotypes scattered between sample types with conservation of major variants. In addition, no differences in intra- and inter-population encoded protein variability were found between the different types of isolates or when these were compared to plasma viral RNA within subjects. Additionally, *in vitro* experiments demonstrated phenotypic similarities in terms of replicative capacity and co-receptor usage between viral isolates and plasma viral RNA.

**Conclusion:**

This study is the first in-depth comparison and characterization of viral isolates from different sources and plasma circulating quasispecies using DPS in recently HIV-1 infected subjects. Our data supports the use of primary isolates regardless of their plasma or cellular origin to define genetic variability and biological traits of circulating HIV-1 quasispecies.

## Introduction

Human immunodeficiency virus (HIV-1) exhibits a high degree of genetic diversity particularly difficult to characterize due to the complexity of the RNA viral populations. This complexity is associated with factors such as the lack of proof-reading activity of HIV-1 polymerase, the high rate of generation of viral particles, and the recombination and hypermutagenesis process favored by host cellular proteins [1], [2], [3], [4], [5], [6], [7]. Consequently, the HIV-1 population is composed of a swarm of genetically related variants, known as viral quasispecies, which grant the virus with the ability to quickly adapt to various selective pressures. Examples of the rapid adaptive machinery of HIV-1 are the selection of mutations enabling escape from the humoral and cellular host immune responses [8], [9], [10], [11] and the selection of mutations generating resistance to currently available antiretroviral drugs [12]. Therefore, to define the composition of HIV-1 quasispecies and identify virus diversity or variability within a single infected subject or at the population level it is essential to understand the pathogenesis of HIV-1 and design optimal antiretroviral treatments and vaccines.

Some studies associated pathogen diversity with poor prognosis [13], [14], [15], and increased diversity of HIV-1 has been related to disease progression [16], [17]. As a result, the maintenance of virus population structures in primary isolates is a key feature for the accurate study of specific viral biological traits, such as fitness and co-receptor usage, which are central to completing our understanding of the HIV-1 pathogenesis. The recent development of a new generation of massively parallel sequencing technologies has enabled us to carry out comprehensive studies of the genotypic characteristics of viral populations, genetically comparing thousands of sequences and increasing our chances of identifying minority variants. Deep Pyrosequencing (DPS) technology has made possible to describe the complexity of viral dynamics during immune escape, to quantify the presence of minority drug resistance variants, and to define virus co-receptor use for the management of CCR5 antagonists [18], [19], [20], [21], [22].

This study aims to investigate with the use of DPS technologies whether viral isolates from biological samples preserves the variability of circulating viruses and the phenotypic features found *in vivo*. For that reason, we compared paired HIV-1 isolates obtained from plasma and cells with total plasma viral RNA in four recently HIV-1-infected subjects. We combined multiple-amplicon DPS covering *gag*, protease, integrase, and *env*-V3 with *in vitro* replicative capacity and virus co-receptor use assays in order to address the genetic and phenotypic associations between HIV-1 isolates and viral quasispecies.

## Results

### Efficiency of HIV-1 recovery correlates with sample viral load for both plasma-derived and cell-derived viral isolates

**Figure 1:**
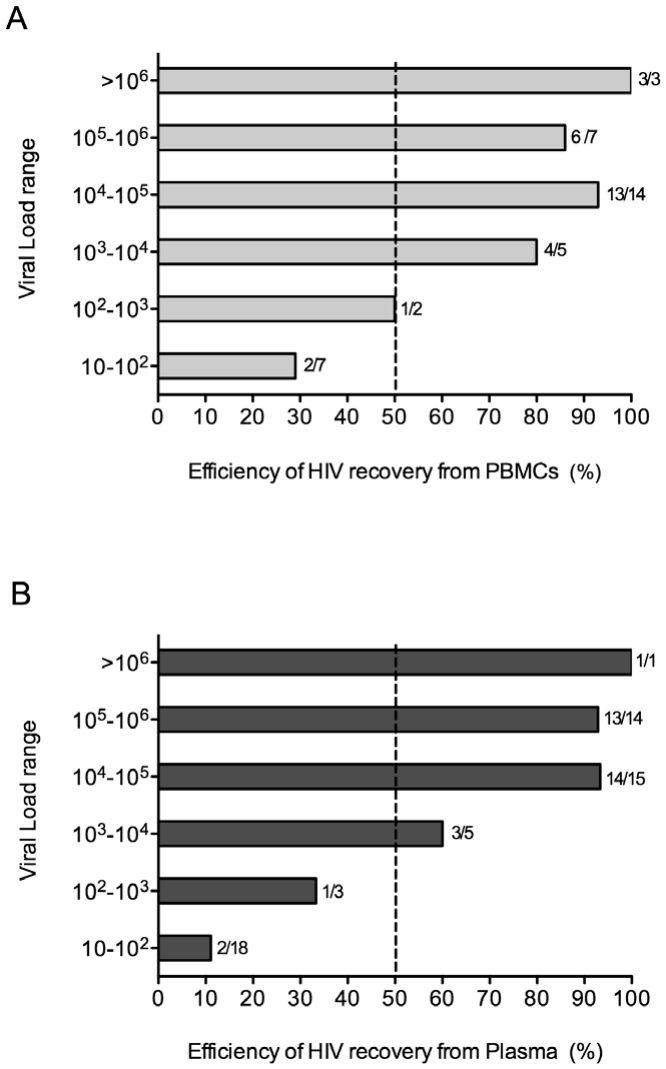
Comparison of the efficiencies of HIV-1 isolation methods from PBMCs or plasma samples. To determine the efficiency of HIV-1 isolation from PBMCs and plasma, we compared virus recovery from 56 plasma samples and 38 PBMCs samples with viral load ranging from 10 to >10^6^ copies/ml. (**A**) Efficiency of HIV-1 recovery from PBMCs in percentages per viral load range. (**B**) Efficiency of HIV-1 recovery from plasma samples in percentages per viral load range. Bars represent mean values. Numbers next to the bars indicate (number of positive samples/total number of samples tested).

In order to compare the efficiency of the methods used to obtain primary HIV-1 isolates from plasma or peripheral blood mononuclear cells (PBMCs), we analyzed a total 94 samples from different subjects at unique time-points, with the exception of the four included in the study; 56 plasma samples and 38 PBMCs samples with viral loads ranging from 10 to >10^6^ copies/ml. Of those, we recovered a total of 63 primary isolates (34 from plasma samples and 29 from PBMCs). After stratification of samples by viral load, we observed an increase in the efficiency of virus recovery concomitant with the increase in viral load for both plasma and PBMCs HIV-1 isolation methods, Fig. 1. Furthermore, the categorization of viral load ranges into linear values demonstrated the existence of a direct correlation between sample viral load range and efficiency of virus recovery (Plasma: r = 0.94, p<0.016; PBMCs: r = 0.94 p<0.016 [Spearman correlation test]). Therefore, overall efficiency of the HIV-1 isolation methods used was similar and correlated to sample viral load.

### Phylogenetic analysis of multiple-amplicon DPS reveals clusters of interspersed variants between cell virus isolates, plasma virus isolates, and plasma viral RNA

**Figure 2:**
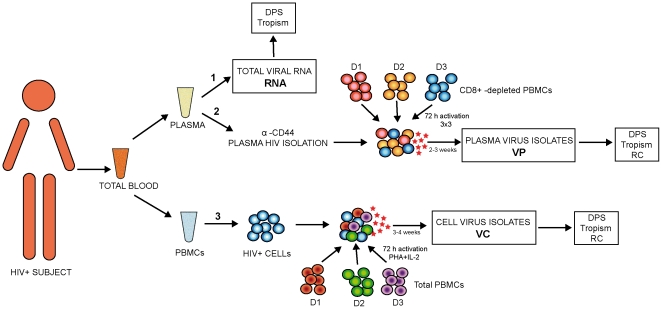
Schematic representation HIV-1 sample types analyzed for comparative purposes per study subject. Total blood was separated into plasma and PBMCs for the following: **1.** Total viral RNA extraction (RNA) used for DPS and virus tropism. **2.** Plasma virus isolation (VP) used for DPS, virus tropism and Replicative Capacity (RC): VP isolates were obtained by mixing plasma extracted anti-CD44 HIV-1 particles with a pool of CD8^+^-depleted PBMCs from three seronegative-donors (D1, D2, and D3) and culture during 2 to 3 weeks for virus *in vitro* expansion. **3.** Cell virus isolation (VC) used for DPS, virus tropism and RC. VC isolates were obtained by co-culture of HIV+ cells with a pool of total PBMCs from three seronegative-donors (D1, D2, and D3) and culture during 3 to 4 weeks for virus *in vitro* expansion. Colored red, orange and dark blue circles represent cells from CD8+ depleted seronegative donors D1, D2 and D3 respectively. Colored red, green and purple circles represent cells from seronegative donors D1, D2 and D3 respectively. Light blue circles represent HIV+ cells. Red stars indicate virus production.

Four naïve, recent HIV-1-infected subjects were enrolled in the study. A summary of their clinical and epidemiological characteristics is shown in Table 1. Three sample types from a unique blood sample were obtained per subject, as represented in Fig. 2, for comparative purposes: 1.Total plasma viral RNA (RNA); 2. Plasma virus isolates (VP) after HIV-1 capture from plasma and virus *in vitro* expansion and; 3. Cell virus isolates (VC) obtained from PBMCs co-culture and virus *in vitro* expansion.

**Table 1:** Epidemiological and clinical data of study subjects.

Subject	Sex	Virus^a^ Subtype	Time after seroconversion (months)	Viral Load at sample collection (HIV-1 RNA copies/ml)	CD4 T-cell count at sample collection (cells/µl)	Nadir CD4 T-cell count (cells/µl)
P20	Male	B	5.5	69,000	190	144
P21	Male	B	13.4	320,000	181	181
P22	Male	BF	4.2	27,000	576	242
P23	Female	B	1.3	170,000	627	197

a Virus subtype was determined based on sequences from *gag*, pol, and *env*-V3 using the REGA HIV-1 Subtyping tool. BF denotes the recombinant BF HIV-1 form.

VP and VC primary isolates were expanded *in vitro* for a period of 2 to 3 weeks and 3 to 4 weeks respectively. Afterwards, virus were harvested for further genotypic (DPS) and phenotypic characterization (Tropism and Replicative Capacity) Fig. 2.

**Figure 3:**
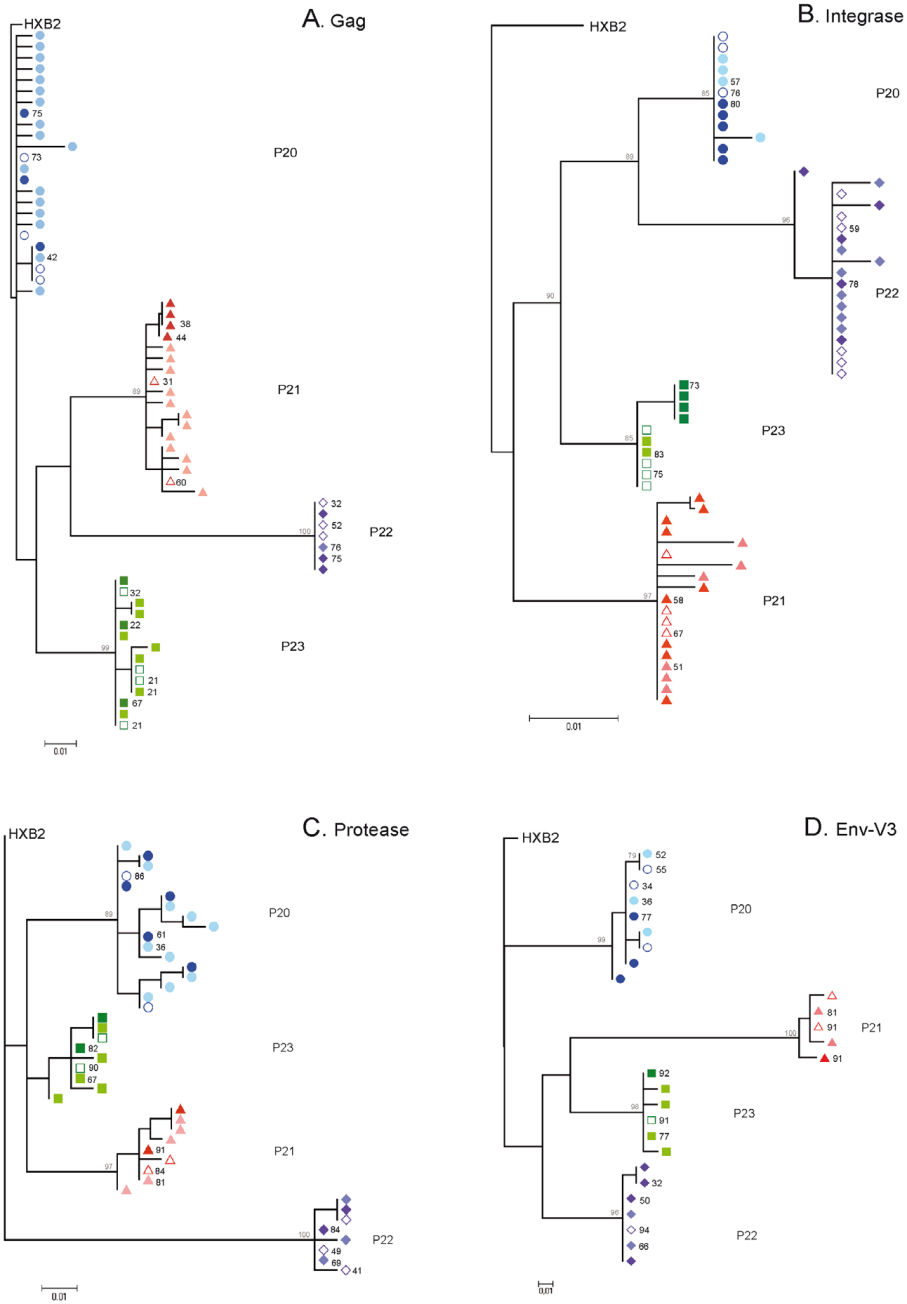
Phylogenetic trees for HIV-1 *gag*, protease, integrase and *env*-V3 sequences extracted from VC, VP, and RNA using DPS. Symbols represent unique haplotypes extracted from DPS for subjects P20 ○, P21 ▵, P22 ⋄, and P23 □, according to sample type: VC (light symbols), VP (dark symbols), and RNA (empty symbols). Numbers next to symbols indicate haplotype frequencies obtained from the total reads; only values above 20% are indicated in the figure. Node numbers indicate bootstrap values over 75%. (**A**) *gag* maximum-likelihood phylogenetic tree based on the TrN model. (**B**) Integrase maximum-likelihood phylogenetic tree based on the HKY model. (**C**) Protease maximum-likelihood phylogenetic tree based on the HKY+ G (α = 0.565) model. (**D**) *env*-V3 maximum-likelihood phylogenetic tree based on the TrN model.

Multiple-amplicon DPS was carried out in the three samples types RNA, VP, and VC, thus covering the *gag*, protease, integrase, and *env*-V3 regions with an average number of reads per nucleotide of 4039, 4193, 3629, and 4488, respectively. Data extracted using DPS were corrected for sequencing errors, filtered to a final number of unique reads, and merged into haplotypes (unique sequences represented in ≥1%), a resume of the sequences obtained after the various filtering steps is represented in Table 2. Final haplotypes were used to build phylogenetic trees based on the best-inferred model for conserved regions *gag*, protease and integrase as well as variable regions *env*-V3 of the HIV-1 proteome. As shown in Fig. 3, the phylogenetic trees for *gag*, protease, and integrase did not show segregation of clusters between VC, VP, and RNA variants, with low genetic distances between sample types and preservation of major variants after *in vitro* culture. A similar tree topology was observed for the variable *env*-V3 loop region, with clear interspersion of major variants from VC, VP, and RNA. A tendency toward clustering of VP was found in the case of P21 for *gag*, P23 for integrase, and P22 for *env*-V3. However, this pattern was not consistent for other genes within the same subjects. In summary, VC, VP, and total plasma viral RNA populations were structured in closely related quasispecies represented by interspersed variants with a low genetic distance between them.

**Table 2:** Number of sequences obtained for each subject and sample type by DPS.

Subject	Protein	Sample	Total Reads^a^	Valid Reads^b^	Unique Haplotypes^c^
P20		RNA	2,979	786	4
	Gag	VC	4,812	197	17
		VP	4,842	1,383	3
		RNA	3,180	2,321	2
	PR	VC	2,374	1,800	10
		VP	2,541	1,726	5
		RNA	6,898	4,271	3
	IN	VC	2,970	2,102	4
		VP	1,640	1,266	5
		RNA	1,823	1,535	3
	Env-V3	VC	4,776	2,951	3
		VP	3,101	2,701	3
P21		RNA	2,425	1,213	2
	Gag	VC	3,850	89	12
		VP	3,875	2,541	4
		RNA	6,503	5,115	2
	PR	VC	5,385	4,315	5
		VP	3,443	2,806	2
		RNA	3,931	2,702	4
	IN	VC	212	120	6
		VP	1,484	899	9
		RNA	3,073	2,511	2
	Env-V3	VC	5,723	3,594	2
		VP	4,265	3,545	1
P22		RNA	2,960	2,370	3
	Gag	VC	5,284	2,238	1
		VP	3,910	3,146	3
		RNA	2,582	2,138	3
	PR	VC	7,605	3,063	3
		VP	1,812	1,443	2
		RNA	8,334	4,527	6
	IN	VC	6,593	4,143	8
		VP	1,301	1,070	5
		RNA	2,698	2,412	1
	Env-V3	VC	10,669	3,476	2
		VP	5,140	4,335	4
P23		RNA	5,707	4,255	4
	Gag	VC	4,585	2,802	7
		VP	3,244	2,748	3
		RNA	4,189	3,640	2
	PR	VC	2,694	2,282	5
		VP	8,019	6,565	2
		RNA	5,752	4,746	4
	IN	VC	2,906	2,387	2
		VP	1,527	1,289	4
	Env-V3	RNA	2,417	2,083	1
		VC	5,534	3,298	4
		VP	4,639	4,180	1

a Total reads is the total coverage of sequences obtained after direct DPS.

b Valid reads are those sequences obtained after cleaning the total reads by selecting unique sequences with >70% homology to HXB2 and manual correction of homopolymer tacks.

c Unique haplotypes are defined by similar sequences represented as a proportion ≥1% from the total unique reads. DPS, deep pyrosequencing; PR, protease; IN, integrase; RNA, plasma viral RNA; VC, cell virus isolates; VP, plasma virus isolates.

### Low intra- and inter-population variability for VC, VP, and RNA variants among HIV-1 proteins

To define in detail VC, VP, and total viral RNA populations, we calculated intra- and inter-population variability, defined as the tendency for individual genomes to vary from one to another in a population. For that purpose, we simulated a viral population by considering the sequences obtained in the DPS run as a sample of the real population. We measured pairwise intra- and inter-population variability according to sample type for each HIV-1 protein and subject. We found low intra-population variability, with values close to zero for VC, VP, and RNA populations in all subjects and genetic regions (Table 3). Additionally, inter-population analyses comparing RNA with VC, RNA with VP, and VC with VP (Table 3) demonstrated a similar pattern of low variability. These results indicated that VC, VP, and plasma viral RNA populations were composed of HIV-1 variants with low intra- and inter-population variability.

**Table 3:** Comparison of intra- and inter-population variability for each subject, HIV-1 protein, and sample type (RNA, VC, or VP).

		Π^a^_intra_			Π_inter_		
Subject	Protein	Π_int_ RNA	Π_int_ VC	Π_int_ VP	RNA vs VC	RNA vs VP	VC vs VP
P20	Gag	0.0011	0.0110	0.0008	0.0010	<0.0001	0.0009
	PR	0.0005	0.0075	0.0041	0.0022	0.0033	0.0002
	IN	0.0005	0.0013	0.0004	<0.0001	<0.0001	<0.0001
	Env-V3	0.0051	0.0050	0.0018	<0.0001	0.0037	0.0035
P21	Gag	0.0240	0.0300	<0.0001	0.0037	0.0023	0.0040
	PR	0.0006	0.0010	<0.0001	<0.0001	<0.0001	<0.0001
	IN	0.0009	0.0025	0.0014	<0.0001	<0.0001	<0.0001
	Env-V3	0.0002	0.0006	<0.0001	<0.0001	0.0194	0.0194
P22	Gag	<0.0001	<0.0001	<0.0001	<0.0001	<0.0001	<0.0001
	PR	0.0033	0.0018	0.0009	0.0013	0.0012	<0.0001
	IN	0.0009	0.0016	0.0005	<0.0001	<0.0001	0.0002
	Env-V3	<0.0001	<0.0001	0.0437	<0.0001	0.0033	0.0033
P23	Gag	0.0023	0.0036	<0.0001	<0.0001	0.0007	0.0012
	PR	0.0002	0.0029	0.0010	<0.0001	<0.0001	<0.0001
	IN	0.0004	0.0002	0.0006	<0.0001	0.0042	0.0042
	Env-V3	<0.0001	0.0012	<0.0001	<0.0001	<0.0001	<0.0001

a Average number of nucleotide differences per site between sequences. PR, protease; IN, integrase; RNA, plasma viral RNA; VC, cell virus isolates; VP, plasma virus isolates.

### VC and VP isolates display similar *in vitro* replicative capacity in primary cultures

**Figure 4:**
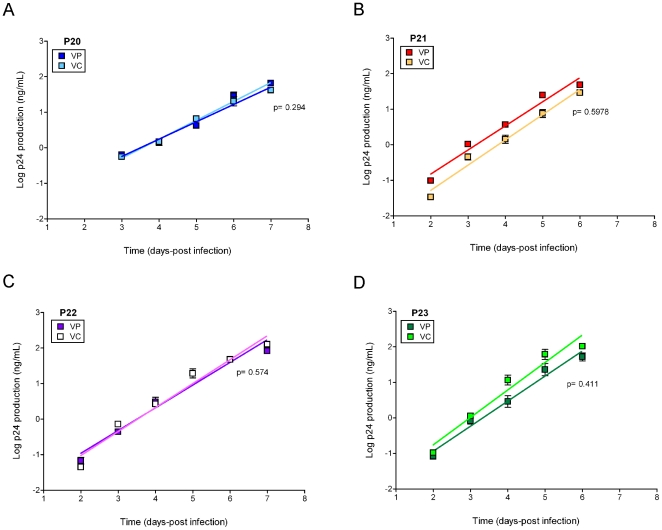
Replicative capacity of VC and VP HIV-1 isolates in primary cells. PBMCs stimulated from seronegative donors were infected in triplicate with each viral variant, and virus growth was monitored by p24 production over one week. Slopes were compared for VC and VP pairs, and p<0.001 was considered significant. Squares represent mean values and bars represent the standard error of the mean. Light squares correspond to VC and dark squares correspond to VP. (**A**) P20, (**B**) P21, (**C**) P22, and (**D**) P23.

In certain cases, heterogeneity in the distribution of quasipecies during *in vitro* passage of HIV-1 modified virus fitness in the absence of changes in the consensus sequences [23]. In order to test whether minor genetic changes described in our populations (synonymous changes, differences in the number of unique variants) affected the phenotypic properties of VC and VP isolates, we measured replicative capacity for VC and VP isolates in primary cells. After infection, viral growth was monitored by p24 production for one week and the log-transformed data on the exponential growth phase used to calculate the virus growth rate (slope of the linear regression) for each type of isolate. As shown in Fig. 4, VC and VP pairs display similar replication kinetics with no differences in replicative capacity per pair in any of the study subjects. Thus, in spite of minor genetic differences in quasispecies composition, our data revealed no differences in replicative capacity between VC and VP isolates for each subject.

### Phenotypic determination and genotypic prediction of co-receptor usage in VC isolates, VP isolates, and plasma RNA

HIV-1 co-receptor use is a key determinant of viral pathogenesis; the presence of CXCR4 using strains has been related to disease progression, and detection of minor CXCR4 variants has a clear clinical interest in the management of CCR5-antagonists [21], [24], [25], [26]. Therefore, in order to understand the relationship between VC, VP and plasma RNA, we compared virus co-receptor use by means of U87 cells in VC and VP isolates and by means of ESTA in plasma RNA. Furthermore, for genotypic prediction of virus co-receptor, we used the PSSM and g2p algorithms in *env*-V3 loop from the most frequent haplotypes, with cut-off values of −4.75 and ≤3.5, respectively. These results are summarized in Table 4. Phenotypic data show a concordance of 100% between U87 and ESTA results. Moreover, genotypic prediction of co-receptor usage with PSSM was 75% (9/12) concordant with g2p. In spite of minor discrepancies between the methods used, plasma RNA, VC, and VP isolates exhibited good matching in terms of virus co-receptor per study subject and sample type. Additionally, a more detailed prediction of co-receptor use was made in VC, VP, and RNA by inference of g2p and PSSM scores in the unique *env*-V3 sequences extracted from DPS. We observed a cluster of combined variants from VC, VP, and RNA with low intra-patient deviation and preferential R5 use, with the exception of p20 Fig. S1. In the case of P20, g2p and PSMM scores from DPS sequences suggest the presence of a homogeneous population of X4R5 dual tropic virus when compared to previously defined R5+X4R5 or X4R5 HIV-1 isolates Fig. S1 [27]. Therefore, inference of phenotypic and genotypic tropism in VC and VP pairs and plasma viral RNA demonstrated concurrence in virus co-receptor usage among sample types for each study subject.

**Table 4:** Phenotypic and genotypic prediction of co-receptor usage from total plasma RNA, VC, and VP primary isolates.

Sample	Phenotype		Genotype	
	U87	ESTA^a^	PSSM^b^	g2p^b^
P20 RNA	-	X4/R5	R5	Non-R5
VC	X4/R5	-	R5	Non-R5
VP	X4/R5	-	R5	Non-R5
P21 RNA	-	R5	R5	R5
VC	R5	-	R5	R5
VP	R5	-	R5	R5
P22 RNA	-	R5	R5	R5
VC	R5	-	R5	R5
VP	R5	-	R5	R5
P23 RNA	-	R5	R5	R5
VC	R5	-	R5	R5
VP	R5	-	R5	R5

a ESTA, Enhance Sensitivity Trofile Assay. This assay has a detection limit of 0.3% for non-R5 variants.

b Cut-off values to define non-R5 using sequences were −4.75 for PSSM and ≤3.5 for g2p, respectively. Non-R5 sequences include X4 and X4/R5 dual tropic virus. RNA, plasma viral RNA, VC, cell virus isolates; VP, plasma virus isolates. – Indicates non-determined.

## Discussion

Primary viral isolates play a key role in our understanding of the HIV-1 pathogenesis and are a common approach for various *in vitro* studies such as antibody neutralization, drug testing, or virus co-receptor use assays. Furthermore, the relevance of using primary isolates for an accurate description of virus phenotype has been highlighted by differences in replicative capacity found between recombinant viruses and full isolates [28], [29].

Previous studies in the HIV-1 field have determined whether viral populations from primary isolates were representative of *in vivo* findings with contradictory results. Some of them report a decrease in HIV-1 gp120 diversity in isolates [30], while others support the maintenance of major variants in blood after co-culture with PBMCs [31]. Additionally, most of these studies are limited by the number of samples analyzed, the number of clonal sequences obtained, and their focus on comparing proviral DNA to primary isolates recovered from co-cultured PBMCs. In order to overcome previous experimental limitations, we carried out multiple-amplicon DPS to genetically compare thousands of sequences in four regions of the HIV-1 genome and clearly define phylogenetic relationships between primary isolates obtained from VP, VC after *in vitro* HIV-1 expansion and plasma circulating quasispecies (RNA) *in vivo*. Our results demonstrate a structured population of interspersed major VC, VP, and RNA variants with fluctuations in low frequency unique sequences in most of the HIV-1 genes studied (*gag*, protease, integrase, and *env*-V3) and among subjects but with no significant differences in the total numbers of unique haplotypes (data not shown). The presence of major variants in similar frequencies for VC and VP primary isolates, when compared to *in vivo* RNA, demonstrates the maintenance of high frequency variants after *in vitro* expansion in both VC and VP isolates. Furthermore, the low intra- and inter-population variability, with values close to zero, reflects homogeneous populations both within HIV-1 proteins or sample types. Nevertheless, relative homogeneous viral populations have been reported in both proviral HIV-1 DNA and plasma HIV-1 RNA during early infection [16], [32]. As a consequence, the low level of genetic variability found among primary viral isolates and total RNA, could be related to the short time after seroconversion in our samples, where homogeneous viral populations will be present before diversification at later stages of disease [16]. On the other hand, recent studies on founder virus evolution support early variation in the HIV-1 genome after transmission and accumulation of changes over the first year after infection [33]. In this context, our results suggest an adequate representation of RNA circulating quasispecies after HIV-1 *in vitro* expansion. However, these results should be viewed with caution until they are confirmed in chronically infected samples.

RNA virus populations are composed of a swarm of closely related genotypes or quasispecies in which viral evolution operates as a unit and adaptation is the result of cooperative interactions between multiple genomes [15]. Various studies have demonstrated how minor genetic differences in composition and quasispecies heterogeneity can modulate HIV-1 fitness in the absence of changes in population sequence [23], [34]. Additionally, genetic similarities in studied regions cannot be extrapolated to the whole viral genome. Therefore, similarities in virus genotype might not take the form of similarities in virus phenotype. In this context, our data revealed no differences in terms of virus replicative capacity in paired VC and VP isolates, regardless of minor differences in genotypic composition of the viral quasispecies studied. However, our approach is limited by the short-term *in vitro* culture of the replicative capacity experiments and presence of antiretroviral drugs, neutralizing antibodies, cytotoxic T lymphocytes, or other selective pressures may induce unpredictable fluctuations in closely related viral populations, which are not capture in this study.

Together with replicative capacity, HIV-1 co-receptor use is an essential trait when defining HIV-1 pathogenesis. The presence of CXCR4-using HIV-1 variants is associated with disease progression [24], [25], [26], and detection of minor CXCR4 HIV-1 populations has become a key marker for the management of CCR5 antagonists [21], [35]. A previous study showed high concordance of co-receptor usage in paired plasma and PBMCs samples during primary infection [36]. In agreement with this observation, we found concordance in co-receptor use between VC isolates, VP isolates, and plasma RNA as measured both by ESTA and U87. Comparable results were obtained by genotypic inference of virus co-receptor use in DPS *env*-V3 sequences with g2p and PSSM. Regardless of small differences in the methods applied intra-subject, co-receptor use was very homogeneous.

Many studies have described the use of DPS in combination with genotypic algorithms in the *env*-V3 variable region as a key tool when detecting minor CXCR4 populations for the management of CCR5 antagonists. We used the same approach to compare VC isolates, VP isolates, and circulating plasma quasispecies. We found clusters of mixed sequences from VC, VP, and RNA sequences with homogeneous populations and preferential R5 use. These data contrast with those of previous studies, where DPS revealed the presence of more heterogeneous populations in proviral quasispecies [35], but argue in favor of homogeneous replication-competent populations obtained after *in vitro* expansion in VC primary isolates, regardless of the heterogeneity in proviral DNA.

In summary, our study provides the first direct comparison of viral isolates with plasma circulating quasipecies using DPS in recently HIV-1 infected subjects. Our data demonstrated that VC and VP share genotypic characteristics with HIV-1 quasispecies and maintain the presence of major variants after virus *in vitro* expansion. In spite of minor genetic differences, phenotypic data reveal similarities in paired VP and VC isolates with regard to replicative capacity and co-receptor use. Our data support the potential use of VP or VC primary isolates as a reliable tool to characterize the circulating quasispecies. Nevertheless, further comparisons will help to clarify whether our findings also apply to later stages of the disease.

## Methods

### Study subjects and Ethics Statement

The study sample comprised four treatment-naïve HIV-1-infected subjects. Epidemiological and clinical data are summarized in Table 1. Virus subtype was assigned based on *gag*, pol, and *env*-V3 sequences using the REGA HIV-1 Subtyping tool. The study was approved by the institutional review board of Hospital Germans Trias i Pujol, and all four subjects gave their written informed consent to participate.

### Cell lines

The following reagents were obtained through the NIH AIDS Research and Reference Reagent Program, Division of AIDS, NIAID, NIH: TZM-bl from Dr. John C. Kappes, Dr. Xiaoyun Wu and Tranzyme Inc; U87CXCR4 and U87CCR5 from Dr. HongKui Deng and Dr. Dan R. Littman as previously described [37], [38].

### Plasma virus isolation

Viral isolates were obtained from plasma samples using anti-CD44 beads following the manufacturer's protocol (Miltenyi Biotec, Germany) with minor modifications as previously described [29]. Briefly, before virus extraction, PBMCs from three HIV-1-seronegative donors were isolated and CD8^+^ T cells depleted using the RosetteSep human CD8+ depletion cocktail (Stemcell Technologies, France). Pooled CD8^+^-depleted PBMCs were then stimulated under three different conditions (‘3×3’ method, Miltenyi Biotech). After 72 hours, cells were mixed to a final concentration of 10^6^ cells/ml in R10 supplemented with IL-2 (100 U/ml) (Roche, Spain), and 200 µl of the extracted virus was added to the culture. Cultures were fed weekly with 10^6^ cells/ml fresh 3×3-stimulated cells. Viral growth was monitored weekly using p24 enzyme-linked immunosorbent assay (ELISA) (Innogenetics, Spain). Virus isolates were harvested when the p24 concentration in the supernatant reached at least 100 ng/ml and then stored at −80°C.

### Cell virus isolation

Viral isolates from cryopreserved cells were obtained by co-culture of PBMCs from each HIV-1-infected subject with a pool of PBMCs from three HIV-1-seronegative subjects that had been previously stimulated with phytohemagglutinin (PHA) (3 µg/ml) and IL-2 (10 U/ml) for 72 hours. Viral growth was monitored weekly by p24 ELISA and cultures were fed weekly with fresh cells. Viral stocks were harvested and stored at −80°C.

### PCR amplification and amplicon preparation

Total viral RNA was extracted (QIAamp Viral RNA Mini Kit™, QIAGEN, CA) from plasma (2 ml), plasma viral isolates (1 ml), and cell viral isolates (1 ml) in order to carry out PCR amplification. *gag*, pol, and *env*-V3 were amplified using one-step reverse transcriptase polymerase chain reaction (RT-PCR) (SuperScript® III One-Step RT-PCR System with Platinum® Taq High Fidelity, Invitrogen, Carlsbad, CA, USA) based on a primer set containing 5′-GCA GAA TGG GAT AGA TTG CAT CCA-3′ (1,417→1,440, HXB2) and 5′-CCT TGT TAT GTC CTG CTT GAT ATT CAC-3′ (5,438←5,464, HXB2), and 5′-TAG AGC CCT GGA AGC ATC CAG GAA G-3′ (5853→5877, HXB2) and 5′-TTG CTA CTT GTG ATT GCT CCA TGT-3′ (8,913←8,936, HXB2) for *gag*, pol, and *env*-V3, respectively. Amplification conditions were as follows: 30 minutes at 52°C during reverse transcription, 2 minutes at 94°C, 30 seconds at 94°C, 30 seconds at 55°C, and 4 minutes at 68°C for 25 cycles. A final polymerization step of 5 minutes at 68°C was applied. The enzyme used for the RT-PCR was the Super-Script III one-step PCR (Invitrogen, USA). Amplicons for QDS were generated using carried 454 adaptor A and subject-specific multiple identifiers; pyrosequencing was unidirectional. The conditions for the enzyme were 5 minutes at 94°C, 30 seconds at 52°C, and 1 minute at 68°C for 25 cycles. A final polymerization step of 5 minutes at 68°C was applied. The enzyme used was Platinum High Fidelity (Invitrogen, USA). The specific primer set was composed of the forward primers 5′-CAG GAT TTA AAC ACC ATG CTA AA-3′ (1,333→1,355 HXB2), 5′-AAT TTG CCA GGA AGA TGG-3′ (2,361→2,378 HXB2), 5′-TTA AGG CCG CCT GTT G-3′ (4,606→4,621 HXB2), and 5′-TGG CAG TCT AGC AGA AGA AG-3′ (7,010→7,029 HXB2), and the reverse primers 5′-TAT CCA TCT TTT ATA GAT TTC TCC-3′ (1,564←1,587 HXB2), 5′-CAA TAG GAC TAA TGG GAA AA-3′ (2,546←2,565 HXB2), 5′-TTT TGT AAT TTG TTT TTG TAA TTC-3′ (4,863←4,886 HXB2), and 5′-CTG GGT CCC CTC CTG AGG-3′ (7,315←7,332 HXB2) for *gag*, protease, integrase, and *env*-V3, respectively. All PCR reactions were performed in triplicate to reduce amplification bias and the founder effects. Triplicate amplifications were pooled before the purification procedure. Reactions were purified using the Agencourt AMPure Kit (Beckman Coulter, Germany) to eliminate the primer-dimers produced. The number of molecules was quantified by fluorometry using the Quant-iT PicoGreen dsDNA assay kit (Invitrogen, USA). When concentrations were below 5 ng/ml, amplicon quality was assessed by spectrometry using BioAnalyzer (Agilent Technologies, USA). Quantitative multiple amplicon DPS was performed in a 454 Genome Sequencer FLX (454 Life Sciences/Roche, USA) using FLX chemistry. A pNL4.3 clone was sequenced to assess the likelihood of errors during DPS. Discrepancies between data obtained by DPS and Sanger sequencing of pNL4.3 clone were attributed to the process.

### Multiple amplicon DPS data clean-up and phylogenetic analysis

Data were cleaned in order to increase the quality of the sequences for down-stream analysis after multiple-amplicon DPS. The first step was to retrieve those sequences with a similarity >70%, when compared with HXB2 from the sequencing run. We then manually corrected the homopolymer tracks, since these are the most common sequencing errors produced by the technique. Sequences with stop codons within the open reading frame of the protein were removed from the analysis, and sequences containing gaps were maintained and included in the analysis, rather than being removed using a conservative bias towards an unknown nucleotide at this position. Identical sequences were collapsed into a single unique sequence or haplotype. Haplotypes with less than 1% presence in the population were removed from the analysis. A summary of the number of reads after the various filtering steps and the final number of haplotypes is represented in Table 2. Phylogenetic trees were built on the nucleotide alignment for the total unique reads collapsed into unique haplotypes. The best phylogenetic model was inferred using jModeltest v0.1.1 [39] for each HIV-1 protein in all subjects. Phylogenetic trees were constructed taking into account the inferred model in PhyML over 1000 bootstrap replicates (www.HIV-1.lanl.gov) were: for *gag* (TrN), protease (HKY+G), integrase (HKY) and *env*-V3 (TrN). Newick trees were exported and edited with MEGA4 [40].

### Population variability per HIV-1 protein and sample type

To study and reproduce the variability according to sample type and among HIV-1 proteins, we simulated a viral population taking into account the sequences obtained in the sequencing run as a sample of the real population. The percentage of each sequence, based on the sequencing run, was used to create a population of 100 sequences where each haplotype was represented as many times as indicated by the percentage of the sequence in the sequencing run. This population of 100 sequences was used to infer variability among populations in the same patient and among HIV-1 proteins. We measured pairwise intra- and inter-population variability using the best model found by jModeltest v0.1.1, as implemented in MEGA4.

### Replicative capacity experiments

Viral isolates obtained from plasma and cells were titrated in the TZM-bl immortalized cell line. Replicative capacity experiments were carried out using PBMCs from three seronegative individuals; previous infection PBMCs were stimulated for 72 hours with PHA (3 µg/ml) and IL-2 (10 U/ml). Stimulated PBMCs were then infected in triplicate with an equal multiplicity of infection of each viral variant at 37°C for 2 hours. Pellets were washed twice with phosphate-buffered saline (PBS) and cultured at 37°C and 5% CO_2_ in R20 supplemented with IL-2 (20 U/ml) (Roche, Spain) [41]. Viral growth was measured by p24 ELISA in supernatants over 10 days (Perkin Elmer, Spain). Replicative capacity was calculated by fitting a linear model to the log_10_-transformed data of p24 production and comparing the slopes as previously described [42].

### Determination of virus co-receptor use

Viral tropism from VP and VC was measured in U87 immortalized cell lines expressing CCR5 or CXCR4, as previously described [27], [38]. Briefly, 5,000 cells were plated on a 96-well plate and infected with 2 ng of p24 for each viral variant overnight. The next day, virus was washed 3 times with 200 µl of PBS and fresh media added to a final volume of 200 µl. Five days after infection, virus growth was identified microscopically by observation of syncytium formation, and the results were corroborated by p24. Furthermore, virus tropism was assessed in plasma samples at similar time-points using the Enhance Sensitivity Trofile Assay (ESTA, with a detection limit of 0.3% for non-R5 variants). In addition, two algorithms were used to infer virus co-receptor use based on *env*-V3 loop sequences from DPS: PSSM (http://indramullins.microbiol.washington.edu/webpssm) and geno2pheno (g2p) (http://www.geno2pheno.org/) with a false positive rate of 10%. Cut-off values to define non-R5 using sequences were −4.75 for PSSM and ≤3.5 for g2p [21].

## Supporting Information

Figure S1 **Genotipic predicition of co-receptor use in DPS sequences from VC, VP and total plasma RNA.** Unique sequences obtained from the DPS of the *env*-V3 loop region were used to run PSSM and g2p algorithms to infer virus co-receptor use per sample type VC (ligth symbols), VP (dark symbols) and RNA (empty symbols) and subject. For comparative purposes *env*-V3 loop sequences from virus with dual mix (R5+R5X4) and dual (R5X4) co-receptor use were included. (**A**) Prediction of co-receptor use based on g2p algorithm with a false positive rate of 10%. Dashed line represents cut-off values (3.5) to infer R5 and non-R5 use. (**B**) Prediction of co-receptor use based on PSSM scores. Dashed line represents cut-off value (−4.75) to infer R5 and non-R5 use.
(TIF)

## References

[1] Harris RS, Bishop KN, Sheehy AM, Craig HM, Petersen-Mahrt SK (2003). DNA deamination mediates innate immunity to retroviral infection.. Cell.

[2] Ho DD, Neumann AU, Perelson AS, Chen W, Leonard JM (1995). Rapid turnover of plasma virions and CD4 lymphocytes in HIV-1 infection.. Nature.

[3] Mangeat B, Turelli P, Caron G, Friedli M, Perrin L (2003). Broad antiretroviral defence by human APOBEC3G through lethal editing of nascent reverse transcripts.. Nature.

[4] Perelson AS, Neumann AU, Markowitz M, Leonard JM, Ho DD (1996). HIV-1 dynamics in vivo: virion clearance rate, infected cell life-span, and viral generation time.. Science.

[5] Preston BD, Poiesz BJ, Loeb LA (1988). Fidelity of HIV-1 reverse transcriptase.. Science.

[6] Zhang H, Yang B, Pomerantz RJ, Zhang C, Arunachalam SC (2003). The cytidine deaminase CEM15 induces hypermutation in newly synthesized HIV-1 DNA.. Nature.

[7] Wood N, Bhattacharya T, Keele BF, Giorgi E, Liu M (2009). HIV evolution in early infection: selection pressures, patterns of insertion and deletion, and the impact of APOBEC.. PLoS Pathog.

[8] Honeyborne I, Codoner FM, Leslie A, Tudor-Williams G, Luzzi G (2010). HLA-Cw*03-restricted CD8+ T-cell responses targeting the HIV-1 gag major homology region drive virus immune escape and fitness constraints compensated for by intracodon variation.. J Virol.

[9] Phillips RE, Rowland-Jones S, Nixon DF, Gotch FM, Edwards JP (1991). Human immunodeficiency virus genetic variation that can escape cytotoxic T cell recognition.. Nature.

[10] Peyerl FW, Barouch DH, Letvin NL (2004). Structural constraints on viral escape from HIV- and SIV-specific cytotoxic T-lymphocytes.. Viral Immunol.

[11] Wei X, Decker JM, Wang S, Hui H, Kappes JC (2003). Antibody neutralization and escape by HIV-1.. Nature.

[12] Larder BA, Kemp SD (1989). Multiple mutations in HIV-1 reverse transcriptase confer high-level resistance to zidovudine (AZT).. Science.

[13] Irshad M, Ansari MA, Singh A, Nag P, Raghvendra L (2010). HCV-genotypes: a review on their origin, global status, assay system, pathogenecity and response to treatment.. Hepatogastroenterology.

[14] Jerzak GV, Bernard K, Kramer LD, Shi PY, Ebel GD (2007). The West Nile virus mutant spectrum is host-dependant and a determinant of mortality in mice.. Virology.

[15] Vignuzzi M, Stone JK, Arnold JJ, Cameron CE, Andino R (2006). Quasispecies diversity determines pathogenesis through cooperative interactions in a viral population.. Nature.

[16] Shankarappa R, Margolick JB, Gange SJ, Rodrigo AG, Upchurch D (1999). Consistent viral evolutionary changes associated with the progression of human immunodeficiency virus type 1 infection.. J Virol.

[17] Troyer RM, Collins KR, Abraha A, Fraundorf E, Moore DM (2005). Changes in human immunodeficiency virus type 1 fitness and genetic diversity during disease progression.. J Virol.

[18] Codoner FM, Pou C, Thielen A, Garcia F, Delgado R (2010). Dynamic escape of pre-existing raltegravir-resistant HIV-1 from raltegravir selection pressure.. Antiviral Res.

[19] Fischer W, Ganusov VV, Giorgi EE, Hraber PT, Keele BF (2010). Transmission of single HIV-1 genomes and dynamics of early immune escape revealed by ultra-deep sequencing.. PLoS One.

[20] Hedskog C, Mild M, Jernberg J, Sherwood E, Bratt G (2010). Dynamics of HIV-1 quasispecies during antiviral treatment dissected using ultra-deep pyrosequencing.. PLoS One.

[21] Swenson LC, Mo T, Dong WW, Zhong X, Woods CK (2011). Deep sequencing to infer HIV-1 co-receptor usage: application to three clinical trials of maraviroc in treatment-experienced patients.. J Infect Dis.

[22] Li JZ, Paredes R, Ribaudo HJ, Svarovskaia ES, Metzner KJ (2011). Low-frequency HIV-1 drug resistance mutations and risk of NNRTI-based antiretroviral treatment failure: a systematic review and pooled analysis.. Jama.

[23] Borderia AV, Lorenzo-Redondo R, Pernas M, Casado C, Alvaro T (2010). Initial fitness recovery of HIV-1 is associated with quasispecies heterogeneity and can occur without modifications in the consensus sequence.. PLoS One.

[24] Koot M, Keet IP, Vos AH, de Goede RE, Roos MT (1993). Prognostic value of HIV-1 syncytium-inducing phenotype for rate of CD4+ cell depletion and progression to AIDS.. Ann Intern Med.

[25] Richman DD, Bozzette SA (1994). The impact of the syncytium-inducing phenotype of human immunodeficiency virus on disease progression.. J Infect Dis.

[26] Connor RI, Sheridan KE, Ceradini D, Choe S, Landau NR (1997). Change in coreceptor use correlates with disease progression in HIV-1–infected individuals.. J Exp Med.

[27] Dalmau J, Puertas MC, Azuara M, Marino A, Frahm N (2009). Contribution of immunological and virological factors to extremely severe primary HIV type 1 infection.. Clin Infect Dis.

[28] Buzon MJ, Dalmau J, Puertas MC, Puig J, Clotet B (2010). The HIV-1 integrase genotype strongly predicts raltegravir susceptibility but not viral fitness of primary virus isolates.. Aids.

[29] Prado JG, Prendergast A, Thobakgale C, Molina C, Tudor-Williams G (2010). Replicative capacity of human immunodeficiency virus type 1 transmitted from mother to child is associated with pediatric disease progression rate.. J Virol.

[30] Kusumi K, Conway B, Cunningham S, Berson A, Evans C (1992). Human immunodeficiency virus type 1 envelope gene structure and diversity in vivo and after cocultivation in vitro.. J Virol.

[31] Voronin Y, Chohan B, Emerman M, Overbaugh J (2007). Primary isolates of human immunodeficiency virus type 1 are usually dominated by the major variants found in blood.. J Virol.

[32] Gottlieb GS, Heath L, Nickle DC, Wong KG, Leach SE (2008). HIV-1 variation before seroconversion in men who have sex with men: analysis of acute/early HIV infection in the multicenter AIDS cohort study.. J Infect Dis.

[33] Keele BF, Giorgi EE, Salazar-Gonzalez JF, Decker JM, Pham KT (2008). Identification and characterization of transmitted and early founder virus envelopes in primary HIV-1 infection.. Proc Natl Acad Sci U S A.

[34] Yuste E, Borderia AV, Domingo E, Lopez-Galindez C (2005). Few mutations in the 5′ leader region mediate fitness recovery of debilitated human immunodeficiency type 1 viruses.. J Virol.

[35] Abbate I, Rozera G, Tommasi C, Bruselles A, Bartolini B (2010). Analysis of co-receptor usage of circulating viral and proviral HIV genome quasispecies by ultra-deep pyrosequencing in patients who are candidates for CCR5 antagonist treatment.. Clin Microbiol Infect.

[36] Raymond S, Delobel P, Mavigner M, Cazabat M, Encinas S (2010). CXCR4-using viruses in plasma and peripheral blood mononuclear cells during primary HIV-1 infection and impact on disease progression.. Aids.

[37] Platt EJ, Wehrly K, Kuhmann SE, Chesebro B, Kabat D (1998). Effects of CCR5 and CD4 cell surface concentrations on infections by macrophagetropic isolates of human immunodeficiency virus type 1.. J Virol.

[38] Bjorndal A, Deng H, Jansson M, Fiore JR, Colognesi C (1997). Coreceptor usage of primary human immunodeficiency virus type 1 isolates varies according to biological phenotype.. J Virol.

[39] Posada D (2008). jModelTest: phylogenetic model averaging.. Mol Biol Evol.

[40] Tamura K, Dudley J, Nei M, Kumar S (2007). MEGA4: Molecular Evolutionary Genetics Analysis (MEGA) software version 4.0.. Mol Biol Evol.

[41] Villena C, Prado JG, Puertas MC, Martinez MA, Clotet B (2007). Relative fitness and replication capacity of a multinucleoside analogue-resistant clinical human immunodeficiency virus type 1 isolate with a deletion of codon 69 in the reverse transcriptase coding region.. J Virol.

[42] Prado JG, Honeyborne I, Brierley I, Puertas MC, Martinez-Picado J (2009). Functional consequences of human immunodeficiency virus escape from an HLA-B*13-restricted CD8+ T-cell epitope in p1 Gag protein.. J Virol.

