# Supplementary figures and images for: In–Depth Characterization of Viral Isolates from Plasma and Cells Compared with Plasma Circulating Quasispecies in Early HIV-1 Infection
Source: PLoS One. 2012 Feb 29;7(2):e32714. doi: 10.1371/journal.pone.0032714 (PMC3290612; doi:10.1371/journal.pone.0032714)

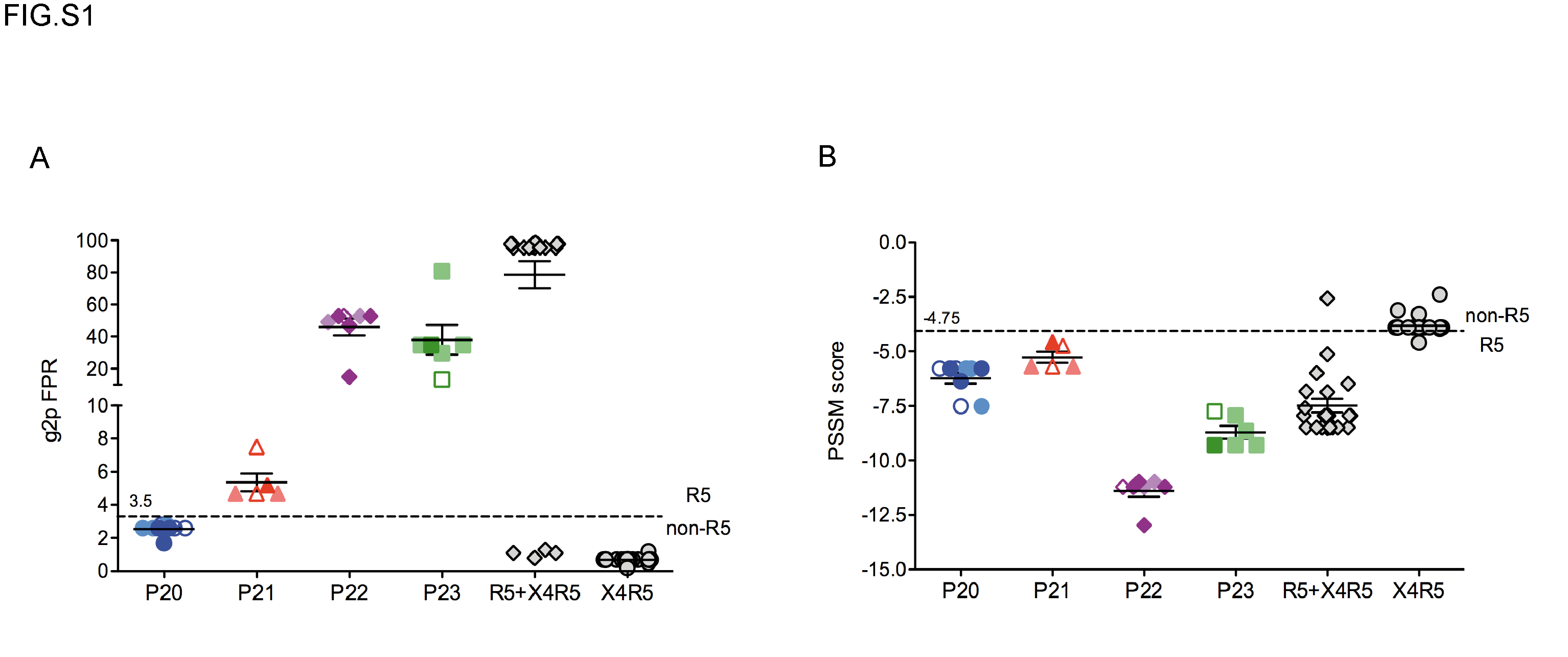

Supplement: Figure S1 — Genotipic predicition of co-receptor use in DPS sequences from VC, VP and total plasma RNA. Unique sequences obtained from the DPS of the env-V3 loop region were used to run PSSM and g2p algorithms to infer virus co-receptor use per sample type VC (ligth symbols), VP (dark symbols) and RNA (empty symbols) and subject. For comparative purposes env-V3 loop sequences from virus with dual mix (R5+R5X4) and dual (R5X4) co-receptor use were included. (A) Prediction of co-receptor use based on g2p algorithm with a false positive rate of 10%. Dashed line represents cut-off values (3.5) to infer R5 and non-R5 use. (B) Prediction of co-receptor use based on PSSM scores. Dashed line represents cut-off value (−4.75) to infer R5 and non-R5 use. (TIF) [file pone.0032714.s001.tif]
